# A comparison of microarray and MPSS technology platforms for expression analysis of Arabidopsis

**DOI:** 10.1186/1471-2164-8-414

**Published:** 2007-11-12

**Authors:** Junfeng Chen, Vikas Agrawal, Magnus Rattray, Marilyn AL West, Dina A St Clair, Richard W Michelmore, Sean J Coughlan, Blake C Meyers

**Affiliations:** 1 School of Computer Science, University of Manchester, Oxford Road, Manchester, M13 9PL, UK; 2 Department of Plant and Soil Sciences, and Delaware Biotechnology Institute, University of Delaware, Newark, Delaware 19714, USA; 3 Plant Sciences Department, University of California-Davis, One Shields Ave, Davis, CA 95616, USA; 4 The Genome Center & the Department of Plant Sciences, University of California-Davis, One Shields Ave, Davis, CA 95616, USA; 5 Agilent Technologies Inc., Little Falls Site, 2850 Centerville Road, Wilmington, DE 19808-1644, USA

## Abstract

**Background:**

Several high-throughput technologies can measure in parallel the abundance of many mRNA transcripts within a sample. These include the widely-used microarray as well as the more recently developed methods based on sequence tag abundances such as the Massively Parallel Signature Sequencing (MPSS) technology. A comparison of microarray and MPSS technologies can help to establish the metrics for data comparisons across these technology platforms and determine some of the factors affecting the measurement of mRNA abundances using different platforms.

**Results:**

We compared transcript abundance (gene expression) measurement data obtained using Affymetrix and Agilent microarrays with MPSS data. All three technologies were used to analyze the same set of mRNA samples; these samples were extracted from various wild type *Arabidopsis thaliana *tissues and floral mutants. We calculated correlations and used clustering methodology to compare the normalized expression data and expression ratios across samples and technologies. Abundance expression measurements were more similar between different samples measured by the same technology than between the same sample measured by different technologies. However, when expression ratios were employed, samples measured by different technologies were found to cluster together more frequently than with abundance expression levels.

Furthermore, the two microarray technologies were more consistent with each other than with MPSS. We also investigated probe-position effects on Affymetrix data and tag-position effects in MPSS. We found a similar impact on Affymetrix and MPSS measurements, which suggests that these effects were more likely a characteristic of the RNA sample rather than technology-specific biases.

**Conclusion:**

Comparisons of transcript expression ratios showed greater consistency across platforms than measurements of transcript abundance. In addition, for measurements based on abundances, technology differences can mask the impact of biological differences between samples and tissues.

## Background

The availability of whole genome sequences and the development of high-throughput technologies have enabled global or genome-wide measurements of gene expression. The complete genome sequence of *Arabidopsis thaliana *[1] is available and this plant serves as an important model for gene expression research. A number of high-throughput technologies have been developed, such as the widely used microarray technologies [2,3] and tag- or sequence-based technologies [4-7], that measure the abundance of many different mRNA transcripts within a biological sample. Several microarray platforms have been developed with probe sequences based on the sequenced Arabidopsis genome. Some of the most recent generation of commercially-produced Arabidopsis microarrays represent more than 21,000 genes: Affymetrix [3] and Agilent Technologies. [8]. The Affymetrix microarray contains sets of 25-nucleotide probes per gene, while Agilent utilizes a single 60-nucleotide probe per gene on the microarray.

Hybridization signal intensities can be used to generate relative or abundance measurements that correspond to the amount of target mRNA that has hybridized to a specific probe, but relative measurements are determined by a comparison of two samples. Tag-based technologies measure the expression level of a gene by counting the abundance of a specific transcript in a sample. This count provides an abundance measure of each gene's expression level within the sample. Until recently, only two tag-based technologies have been widely used: SAGE (Serial Analysis of Gene Expression) [4,5] and MPSS (Massively Parallel Signature Sequencing) technology [6,7]. Both SAGE and MPSS produce short (10–22 nucleotide) sequence tags that are derived from a defined position in a mRNA molecule. A significant advantage of the MPSS technology relative to SAGE is the large number of distinct signatures (more than 1,000,000) that can be identified in a single analysis. Therefore, MPSS potentially provides a greater coverage of the transcriptome than SAGE.

Previous studies have compared different gene expression measurement technologies to determine how well they correlate with each other and/or to use one technology as a benchmark for another [9-24]. Some early studies, comparing different microarray platforms, showed that commercial microarrays were typically more consistent than non-commercial microarrays [11,13,18]. The study of Kuo et al. [18], which compared data from ten different microarray platforms for mouse, found that results were more similar between laboratories using the same platform than across platforms, that one-dye platforms were typically more consistent than two-dye platforms. Recently, some studies have compared microarray and MPSS technology, showing that there is moderate correlation between the two platforms but that one technology will often detect expression for some genes that are not measured by the other platform [22-24]. Oudes et al. [22] therefore suggested that using a combination of transcription profiling technologies would provide more complete coverage of gene expression measurements. Liu et al. [23] provided a comparison between many different microarrays and MPSS technology using mouse tissues but they did not consider as broad a range of biological samples for comparison as in our study.

In a preliminary study, Coughlan et al. [24] compared microarray and MPSS data from *Arabidopsis thaliana*. Here, we extend this preliminary work by carrying out a more detailed data analysis and by including data obtained with the Affymetrix platform. We compare transcript abundance measurements obtained with two types of microarrays (Affymetrix and Agilent technologies) and with MPSS. These data were generated from the same set of mRNA samples extracted from a variety of *Arabidopsis thaliana *tissues, mutants and treatments. We investigate the consistency of transcript abundance measurements across the three technology platforms using comparison of abundance expression levels and expression level ratios. We discuss factors which may affect the measured abundance and contribute to variation in measurements across different technology platforms. This study uses a broad range of samples in order to assess these different platforms.

## Results and Discussion

### Comparison of Gene Expression Data Obtained from MPSS and Microarrays

We believe that there should be some biologically meaningful relationships among different samples from different technology platforms. For example, samples from same tissues with different condition/treatment are expected to have higher correlation than samples from different tissues, and the same samples measured by different technology platforms would be expected to have higher correlation than others. To test our hypothesis, we make comparisons of abundance expression level and comparisons of expression level ratios.

#### Comparison of Abundance Expression Levels

**Figure 1:**
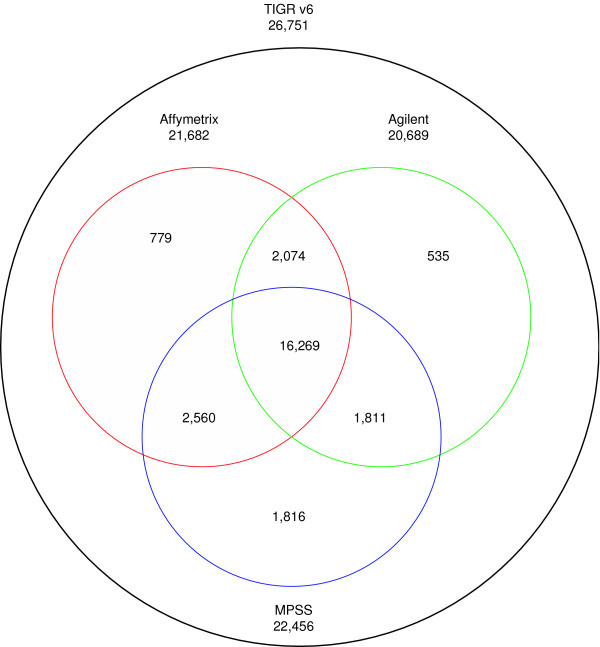
**Protein Coding Genes Potentially Detectable by Technology Platforms**. A Venn diagram indicates the genes (TIGR annotation, version 6) represented on Affymetrix and Agilent microarrays, and detectable by MPSS. The numbers in the overlapping regions of the circles indicate the numbers of genes shared across platforms.

We wanted to compare the abundance of transcripts as measured by the expression data from MPSS, Affymetrix and Agilent technologies (Table 1). It is important to note that not all genes were measurable with all three technologies (Figure 1), therefore our analyses focused on the subset of genes that were represented on both microarray platforms and contained a *Dpn *II site detectable by MPSS (16,269 genes). The MPSS data showed a wider dynamic range than Affymetrix and Agilent data (Table 2). The standard deviations for MPSS data were greater than those for Affymetrix and Agilent data.

**Table 1:** Data used in comparison of microarrays and MPSS technology platforms.

**#**	**Tissue/Treament**	**MPSS**	**Affymetrix**	**Agilent**
1	Callus	MCas	AFCaA/AFCaB	AGCaA/AGCaB
2	Inflorescence	MIns	AFInA/AFInB	AGInA/AGInB, AGSiInA/AGSiInB
3	Leaf	MLes	AFLeA/AFLeB	AGLeA/AGLeB, AGRoLeA/AGRoLeB
4	Root	MRos	AFRoA/AFRoB	AGRoA/AGRoB, AGRoLeA/AGRoLeB
5	Silique	MSis	AFSiA	AGSiInA/AGSiInB
6	Agamous Inflorescence.	MAgm	AFAgmA/AFAgmB	
7	Ap1-10 Inflorescence.	MAp1	AFAp1A/AFAp1B	
8	Ap3-6 Inflorescence.	MAp3	AFAp3A/AFAp3B	
9	Sup/Ap1 Inflorescence.	MSap	AFSapA/AFSapB	
10	Leaves SA 4 hr.	MS04	AFS04A/AFS04B	
11	Leaves SA 52 hr.	MS52	AFS52A/AFS52B	

The eleven RNA sources and corresponding platform or sample codes used in this paper.

Code names:

first two letters of the code: M-MPSS; AF-Affymetrix; AG-Agilent

middle letters of the code: Tissue/Treatment

last letter of the code: A/B-technical replicates

**Table 2:** Basic statistics of the NEU normalized expression data for 6,501 genes detectable by all three technologies.

**Code**	**Minimum**	**Maximum**	**1st Quartile**	**Median**	**3rd Quartile**	**Std**
MCas	0	13615	0	39.74	138.13	460.66
MIns	0	13783	9.69	36.34	109.03	514.51
MLes	0	30874	7.38	41.84	120.59	699.07
MRos	0	17858	7.68	42.23	124.76	498.47
MSis	0	10614	0	44.54	152.37	387.94

AFCa	4.88	2452.5	43.69	82.95	166.42	214.09
AFIn	4.99	2422.8	51.88	88.06	162.84	206.16
AFLe	6.91	3056.1	43.3	78.11	155.42	249.48
AFRo	3.86	3238.8	37.9	69.95	147.11	271.89
AFSi	5.44	2421	45.92	81.65	158.98	225.03

AGCa	0.47	4229.7	36.48	74.02	150.55	304.25
AGIn	1.01	3345.6	44.8	83.74	162.09	242.15
AGLe	0.75	5092.1	39.17	76.78	160	285.71
AGRo	1.41	3375.4	39.38	77.51	166.92	240.88

Std represents the standard error of the mean.

**Figure 2:**
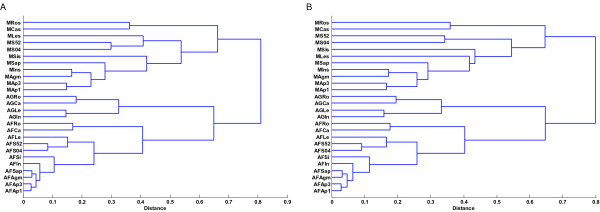
**Hierarchical Clustering of Correlations of Log(NEU) Data for 1,648 Genes in MPSS and Affymetrix**. Plot A shows the Pearson's correlations while Plot B shows the Spearman's rank correlations among the different RNA samples and platforms. Distances for clustering were calculated as 1-r, where r represents correlation coefficient value. A complete linkage algorithm was used for hierarchical clustering. All genes selected for this analysis had a MPSS tag count greater than 4 TPM and were detected as present in Affymetrix microarrays in all eleven samples. Codes for samples are described in Table 1.

To make the data directly comparable, we rescaled the normalized data into Normalized Expression Unit (NEU) values (see Data Analysis in Methods), which we used for abundance expression levels comparison. We calculated the Pearson's correlation and Spearman's rank correlation of logged NEU normalized expression data for 1,648 genes in MPSS, Affymetrix and Agilent (i.e., the genes that were selected to have a MPSS tag count greater than 4 TPM (Transcripts Per Million) and "present" calls determined by the Affymetrix software), and we performed hierarchical clustering using these data (Figure 2). The NEU normalization did not affect the correlation statistics for the logged expression measurements since it corresponds to a shift in the mean for logged data. The within-technology correlations across the tissues and mutants were greater than the inter-technology correlations of identical RNA samples (Table 3). The patterns of clustering and correlation among the samples were similar within technologies, although not identical, when based on either MPSS, Affymetrix or Agilent measurements. Different tissue samples in within-technology comparisons exhibited similar patterns: root was mostly correlated with callus; silique was primarily correlated with flower and secondarily correlated with leaf. The treated leaf samples were clustered together, as were the mutant flower samples.

**Table 3:** Pearson's rank correlation of NEU normalized expression data for 1,648 genes in MPSS, Affymetrix and Agilent.

	MCas	MIns	MLes	MRos	AFCa	AFIn	AFLe	AFRo	AGCa	AGIn	AGLe	AGRo
MCas		0.46	0.41	*0.64*	*0.66*	0.35	0.29	0.50	0.54	0.26	0.19	0.36
MIns			*0.62*	0.56	0.44	*0.71*	0.58	0.50	0.34	0.56	0.42	0.39
MLes				0.50	0.40	0.55	*0.70*	0.43	0.32	0.44	0.53	0.35
MRos					*0.63*	0.51	0.44	*0.76*	0.50	0.39	0.31	0.55

AFCa						*0.68*	*0.60*	**0.83**	*0.74*	0.45	0.35	0.54
AFIn							**0.85**	*0.77*	0.44	*0.69*	0.53	0.51
AFLe								*0.69*	0.4	*0.60*	*0.67*	0.48
AFRo									0.59	0.52	0.41	*0.65*

AGCa										*0.76*	*0.67*	**0.82**
AGIn											**0.85**	**0.82**
AGLe												**0.80**
AGRo												

The upper triangle represents the Pearson's correlation while the corresponding P-values are all smaller than 0.01. The correlation values greater than 0.8 are shown in bold; values between 0.6 to 0.8 are shown in italics. All genes had measurable abundance values greater than 4 TPM in all MPSS samples and were detected as present in Affymetrix.

When comparing the same RNA samples across technologies, the highest correlations were observed for MPSS versus Affymetrix microarrays (0.66 to 0.76) and for Affymetrix microarrays versus Agilent microarrays (0.65 to 0.74) while the lowest correlations were exhibited for Agilent microarrays versus MPSS (0.53 to 0.56) (Table 3). The correlations among different tissues within the MPSS data were lower than the correlations among different tissues measured by Affymetrix and Agilent microarrays. The correlation values among MPSS expression data ranged from 0.41 to 0.64 while the same measurements using Affymetrix or Agilent microarrays ranged from 0.60 to 0.85 and 0.67 to 0.82, respectively. Additional analyses using different Affymetrix and Agilent technical replicates also showed that each pair of technical replicates were closely correlated together, as expected (see Figure 1 in additional file 1, e.g, AFRoA and AFRoB).

**Figure 3:**
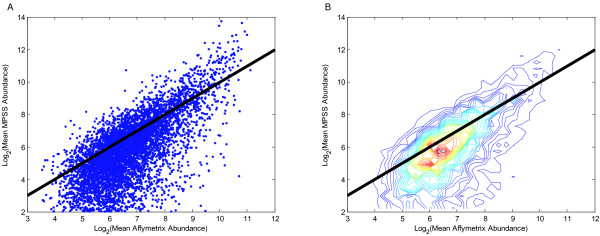
**Underestimation and Overestimation of Transcripts for 6,501 Genes in MPSS and Affymetrix**. Scatter plot (A) and contour plot (B) compare MPSS transcript abundance with Affymetrix abundance; data sets measured with NEU units. All genes selected for this analysis had an MPSS abundance greater than 4 TPM and were detected as present in Affymetrix microarrays. The black bold lines in both plots are the lines representing *x *= *y*. In the contour plot, colors towards red end of the spectrum indicate higher density of data points.

We next assessed the NEU levels from MPSS and Affymetrix technologies in a gene-by-gene comparison of 6,501 genes, which were selected because their mean MPSS abundance across eleven samples was greater than 4 TPM and they were detected as present in the corresponding Affymetrix microarrays for the eleven RNA samples. There were numerous transcripts for which abundance was measurable with the Affymetrix microarrays, but for which no or very little expression was measureable in the MPSS analysis (Figure 3).

#### Comparison of Expression Level Ratios

**Figure 4:**
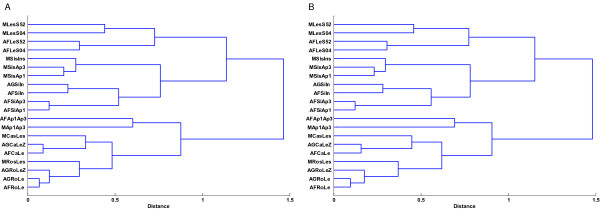
**Hierarchical Clustering of Correlations of Expression Ratios for 1,648 Genes in MPSS, Affymetrix and Agilent**. Plot A shows the Pearson's correlation while Plot B shows the Spearman's rank correlation. Distance for clustering was calculated by 1-r, where r represents correlation coefficient value. A complete linkage algorithm was used for hierarchical clustering. Genes used are the same with those for Figure 2. Note that the Agilent samples with code ending with "Z" are those for which ratio data came from self-self microarray. Codes for samples are described in Table 1.

In our comparison of the MPSS, Affymetrix and Agilent normalized abundance data, we observed that the RNA samples exhibited higher correlations within the technology platform used to measure RNA abundance rather than expected similarities due to the biological nature of the samples. Although MPSS and, in principle, Affymetrix data can be used to estimate abundance expression levels it is likely that probe-specific effects and other technological biases will have a large effect on the estimates. In practice, practitioners often focus on differences in expression between samples. Thus, we decided to use expression ratios of pairwise sample comparisons to reassess the data correlations. Nine samples were selected for this analysis: callus, inflorescence, leaf, root, silique, ap1, ap3, s04 and s52. We used the following eight pairs of samples for calculation of ratios: silique/inflorescence, silique/ap1, silique/ap3, ap1/ap3, callus/leaf, root/leaf, leaf/s04 and leaf/s52. For each technology platform, we computed the Pearson's correlations and Spearman's rank correlations between the ratios across pairs of samples. The hierarchical clustering of the correlations (Figure 4) showed that the samples were more closely clustered according to their biological nature than the technology platform. Therefore, the ratios were a more biologically meaningful measure to use for cluster analysis than abundance measurements of gene expression.

**Figure 5:**
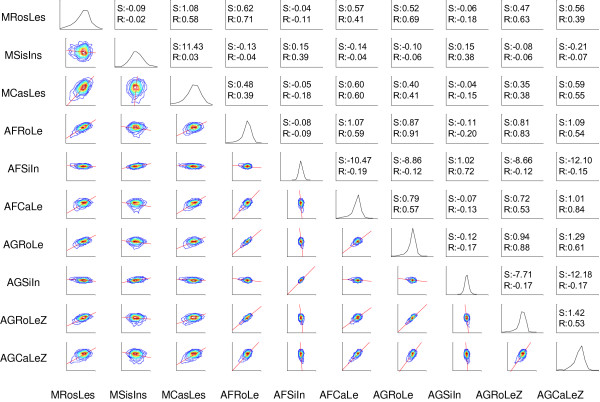
**Expression Ratios for 1,648 Genes in MPSS, Affymetrix and Agilent**. In this matrix type contour plot, the x- and y-axes are ratios of corresponding pairs in MPSS, Affymetrix and Agilent respectively. The axes are on a log-scale. Contours of expression data are shown on the bottom left frames, with red lines representing the total least-square values. Smoothed histograms of the expression data are shown in the diagonal frames. In the top right frames, R is the Spearman's rank correlation value and S is the slope value for the total least square. All data was log-processed. Genes used are the same as those for Figure 2. Codes for samples are described in Table 1.

We next used a contour matrix plot [25] on expression ratios to assess the correlations among the different technology platforms (Figure 5). The Affymetrix and Agilent data were most similar to each other (e.g., AFRoLe and AGRoLe), indicating that the two microarray platforms were more consistent with each other in terms of detection of differential expression than with MPSS. The correlation values using the same tissue pairs for Affymetrix/Agilent and MPSS ranged from 0.38 to 0.71 while the values for Agilent and Affymetrix ranged from 0.72 to 0.91 (Figure 5). We also found that ratios comparing the inflorescence tissue within the different mutants (e.g., MSisAp1/AFSiAp1 and MSisAp3/AFSiAp3) or within leaf tissue from different treatments (e.g., MLesS04/AFLes04 and MLesS52/AFLes52) were not correlated as closely as those between mutants/tissues within the same technology platform (Figure 4). This indicates that comparisons of ratios are not always consistent enough across technologies to identify more subtle differences in a sample. However, there was sufficient consistency to distinguish ratios at a more coarse level (e.g, those involving completely different tissues). Also, the ratio data obtained from the Agilent microarrays which were non-self microarrays (each dye associated with a different sample) with two channels showed higher correlations than the ratio data from self-self microarrays (both dyes associated with the same sample) treated as single-channel microarrays. For example, the Pearson correlation between AFRoLe and AGRoLe was 0.91 while the correlation value between AFRoLe and AGRoLeZ was 0.83. Additional analyses using different Affymetrix and Agilent technical replicates also showed that each pair of ratio data from technical replicates were closely correlated as expected (see Figure 2 in additional file 1, e.g, AFLeS52A and AFLeS52B).

### Probe-position Effects in Affymetrix and Tag-position Effects in MPSS

The position of the microarray probe or MPSS signature within a given transcript may impact the measurement of transcript abundance due to the use of different polyadenylation sites or technical biases [26]. Therefore, we compared the effects of the probe position on the Affymetrix microarray data and the effects of the tag position on the MPSS data. This type of analysis may be useful to distinguish differences in the measured transcript abundances that arise from biological causes in the mRNA sample from those that come from technological biases.

**Figure 6:**
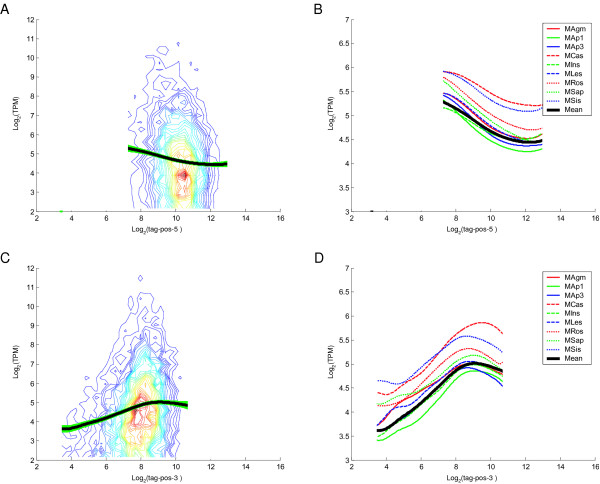
**Tag-position Analysis for 11,932 Genes in MPSS**. All plots compare the measured transcript abundance with the primary tag position. Plots A and B indicate the position of the primary tags calculated from the 5'-end of transcripts, while plots C and D indicate the position of the primary tags calculated from the 3'-end of transcripts. Plots A and C are contour plots of the mean measured abundance across nine MPSS samples, with the bold black curve representing a LOWESS smooth of all the data and the bold green curve representing the bootstrap confidence intervals (5 to 95%). In plots B and D, the bold black lines are the LOWESS smooth of the mean measured abundance, and the remaining lines are the LOWESS smooth of each individual sample. The genes used in this analysis were selected to have abundance data for the primary tag greater than 4 TPM in at least one of the samples. Codes for samples are described in Table 1.

First, we investigated the relationship between the primary tag position and the measured RNA abundance in MPSS. Since MPSS tags are captured via the poly(A) tail and anchored by a four base restriction enzyme recognition site such as GATC, the primary tag was defined as the 3'-most tag in an annotated gene, which was determined based on an analysis of *Dpn *II sites. We analyzed the MPSS data considering the distance of the primary tag from the 5' and 3' ends. We found that primary tags closer to the 3' end tended to have lower measured transcript abundances (Figure 6C and 6D). With increasing distance of the primary tag from the 5' end of the gene, there was a decrease in the MPSS-measured transcript abundance (Figure 6A and 6B).

**Figure 7:**
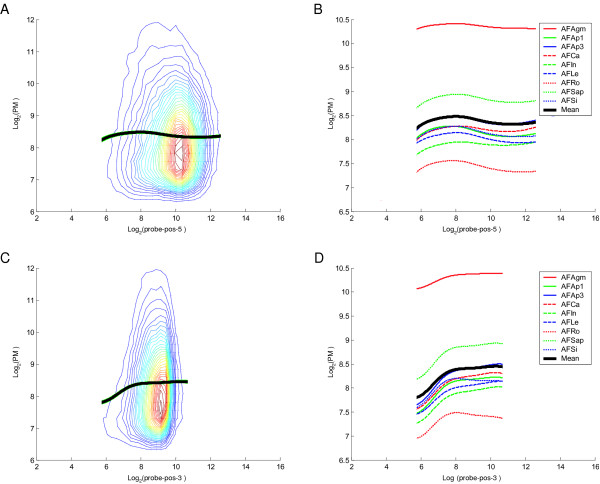
**Probe-position Analysis for 131,239 Probes (corresponding to 11,932 Genes) in Affymetrix**. Plots are similar to those described for Figure 6, except using the probe position for each gene as measured by Affymetrix. The distances of the probes were calculated to both ends of the annotated genes. Codes for samples are described in Table 1.

Next, we investigated the relationship between the probe position and perfect match (PM) probe values in the Affymetrix microarrays. The probe positions were determined relative to the annotated 5' and 3' ends of each gene. We found that the measured transcript abundances correlated with the position from the 3' end such that probes close to the 3' end tended to have lower PM values (Figure 7), but the variation was smaller than for MPSS tags. There was not as strong an association between the measured abundance and distance from the 5' end for the Affymetrix probes compared to MPSS.

Based on the analysis of probe position in the Affymetrix microarrays and the tag position in MPSS samples, we found that both probe position and tag position, as measured from the 3' end, demonstrated a consistent trend. With the increase in probe position or tag position from the 3' end, the PM value and tag-abundance tended to increase then plateau. One reason for this tendency could be that there are many alternative transcripts for some genes which are not correctly annotated in the current Arabidopsis genome which could be alternative splice products or transcripts utilizing alternative polyadenylation sites. In fact, some probes and tags that we used for this analysis were not located within the most recently annotated genome [27,28]. To check whether this tag- or probe-position bias is not merely a consequence of alternative splicing, we were able to obtain similar results when only single exon-containing genes were included (results not shown).

## Conclusion

Our study indicated that the MPSS platform may be more variable in measuring RNA abundance than Affymetrix and Agilent platforms. Our analysis showed that the microarray and MPSS technologies did not correlate well on a quantitative basis for transcript abundance measurements, although within-technology clustering of samples was largely consistent. Expression ratio comparisons were more comparable and consistent across the platform technologies, and ratios for sample pairs involving different tissues were found to correlate quite well across technologies. Therefore, for the purpose of hierarchical clustering of global data sets, the ratios were a more biologically meaningful measure to use for cluster analysis than abundance measurements of gene expression. However, when the difference between the ratios was subtle, as in the case of ratios involving the same pair of tissues but with one derived from a different mutant or treatment, then technological differences often overwhelmed the underlying biological signal. Moreover, the two microarray technologies (Affymetrix and Agilent) were more consistent with each other than with MPSS.

The position of the probe or tag in the transcript has previously been shown to affect the measured transcript abundance [26]. Our analysis demonstrated that there exists a probe-position effect on the abundance measured by Affymetrix, consistent with a similar tag-position effect observed in MPSS data. Specifically, Affymetrix probes and MPSS tags closer to the 3' end of the transcript showed a lower average abundance. The fact that this trend was observed using both technologies suggests that it may reflect an inherent characteristic of the RNA sample. The majority of probes and MPSS tags were within coding regions, and we confirmed that the trend also exists for genes that are annotated as containing only a single exon. This seems to exclude the possibility of alternative splicing as an explanation, unless a significant number of the current gene models are incorrect. One possibility is variation in the polyadenylation site within the 3' UTR. Other possible explanations may be bias in the RNA sample, a misannotation of the coding regions, or a coincidental technological bias shared by Affymetrix microarrays and MPSS.

## Methods

### Data Preparation and Pre-processing

The microarray data and MPSS data for the manuscript has been submitted to the GEO Omnibus [29]. The Series record number is GSE8994 and details relating to sample and data preparation are available there.

Eleven different distinct RNA samples were used in this study (Table 1). The RNA samples were obtained from different *Arabidopsis thaliana *tissues (samples 1 to 5 included callus, inflorescence, leaves, roots and silique), homeotic floral mutants (samples 6 to 9 included *agamous *inflorescence, *apetala1-10 *inflorescence, *apetala3-6 *inflorescence and a *superman/apetala1-10 *inflorescence double mutant) and two leaf treatments (samples 10 and 11 include leaves sampled 4 and 52 hours after salicylic acid treatment, respectively) [24]. Only one technical replication for each sample was available for the MPSS data. Two technical replicates for each sample were available for most of the Affymetrix and Agilent data (codes with last letter "A" or "B"). For the Agilent platform, some microarrays were self-self microarrays (e.g, AFCaA indicates both red and green channels are callus RNA samples) while others are were non-self microarrays (e.g, AGCaLeA indicates that red channel is callus and green channel is leaf RNA sample).

The Arabidopsis MPSS data has been published previously and is available on our website [30]. In the present study, we utilized the "Signature" MPSS samples, rather than "Classic" MPSS samples, because there exists a significant tag-position bias in Classic MPSS samples [26,31]. We also focused on our analyses on the 17-base signatures, rather than the 20-base signatures, because the shorter tags represent a less-biased data set due to a lower frequency of palindromic sequences or other "bad words" [32] that are poorly sequenced by MPSS technology. The raw abundance of each signature in each sample is normalized to a metric of TPM (Transcripts Per Million), which facilitates comparisons across samples and technologies. To obtain the expression level for each gene, we summed the abundance of signatures that match the sense-strand of the gene and are found only once in the Arabidopsis genome and transcriptome.

To generate data that was comparable across technologies, the same Arabidopsis RNA samples used for the MPSS analyses were hybridized to two different high-density Arabidopsis microarray platforms: the Affymetrix ATH1 GeneChip [33] (22,800 features, ~11 probes per gene and 25-nucleotides per probe) and Agilent 22 K Arabidopsis microarray [34] (21,500 features, 60 nucleotide probes and one probe per gene). MAS5.0 software [33] was used to obtain the expression level for each gene on the Affymetrix microarray; Agilent data was preprocessed by Agilent G2566AA Feature Extraction Software [34]. Two technical replicates were obtained for almost all Affymetrix microarrays used to analyze each RNA sample from mutants/treatments/tissues (e.g, AFRoA and AFRoB) and for all Agilent microarrays (e.g, AGRoA and AGRoB which are self-self microarrays and AGRoLeA and AGRoLeB which are non-self microarrays). For the Affymetrix microarray data, for each gene the transcript abundance (expression) measurements from each pair of technical replicates was used to obtain the average (e.g., data was averaged for AFRoA and AFRoB to obtain AFRo). For self-self Agilent microarrays (both dyes associated with the same sample), because each microarray has two-dye channels, for each gene we first averaged the data from the red channel and green channels, then averaged these data from each pair of technical replicates to obtain the final average (e.g., data was averaged for AGRoA and AGRoB to obtain AGRo). Likewise, for Agilent microarrays which were non-self microarrays (each dye associated with a different sample), we averaged the pairs of technical replicates to get the average log ratio (e.g, data averaged for AGRoLeA and AGRoLeB to get AGRoLe).

We downloaded the most recent Arabidopsis genome release data (TIGR version 6, November 2005) from TAIR [35] and focused on the 26,751 protein-coding genes among the total of 31,407 annotated genes. Because each of these microarray platforms and the MPSS platform contained a subset of the total annotated Arabidopsis genes, we compared only the genes represented or detected by all three platforms. A total of 16,269 genes were potentially detectable by all three platforms (Figure 1). For the MPSS data, signatures which were duplicated (i.e., matching multiple genomic locations) were excluded from the analysis; genes matching these signatures were also excluded to avoid a bias that could result if only a subset of the signatures from each gene are used. For the comparison of MPSS and Affymetrix platforms, 6,501 genes were selected since these were detected as present in all eleven Affymetrix microarray data sets and had mean MPSS tag counts across all eleven samples greater than 4 TPM (i.e., the lower identification limit of MPSS signatures [32]). Moreover, for the comparison of the three platforms, 1,648 genes were selected with all MPSS tag counts greater than 4 TPM, detected as present in Affymetrix and detected as present in Agilent. The Agilent ratio data was produced in two different ways: the Agilent ratio data named without "Z" as the last letter was from a non-self microarray, with the log ratio derived from the red channel intensities divided by the green channel intensities; the Agilent ratio data with "Z" as the last letter was from self-self microarrays, with the log ratio obtained from two different microarrays. For example, AGRoLe data is from the root/leaf microarray, while AGRoLeZ data is from root/root and leaf/leaf.

### Data Analysis

To make the data directly comparable, we rescaled the normalized data (see Methods for normalization methods used by each platform) into Normalize Expression Unit (NEU) values, as follows:

Normalized Expression Unit of ith Gene=xi∑j=1nxj×106.
 MathType@MTEF@5@5@+=feaafiart1ev1aaatCvAUfKttLearuWrP9MDH5MBPbIqV92AaeXatLxBI9gBaebbnrfifHhDYfgasaacPC6xNi=xI8qiVKYPFjYdHaVhbbf9v8qqaqFr0xc9vqFj0dXdbba91qpepeI8k8fiI+fsY=rqGqVepae9pg0db9vqaiVgFr0xfr=xfr=xc9adbaqaaeGacaGaaiaabeqaaeqabiWaaaGcbaGaemOta4Kaem4Ba8MaemOCaiNaemyBa0MaemyyaeMaemiBaWMaemyAaKMaemOEaONaemyzauMaemizaqMaeeiiaaIaemyrauKaemiEaGNaemiCaaNaemOCaiNaemyzauMaem4CamNaem4CamNaemyAaKMaem4Ba8MaemOBa4MaeeiiaaIaemyvauLaemOBa4MaemyAaKMaemiDaqNaeeiiaaIaem4Ba8MaemOzayMaeeiiaaIaemyAaK2aaWbaaSqabeaacqWG0baDcqWGObaAaaGccqqGGaaicqWGhbWrcqWGLbqzcqWGUbGBcqWGLbqzcqGH9aqpjuaGdaWcaaqaaiabdIha4naaBaaabaGaemyAaKgabeaaaeaadaaeWbqaaiabdIha4naaBaaabaGaemOAaOgabeaaaeaacqWGQbGAcqGH9aqpcqaIXaqmaeaacqWGUbGBaiabggHiLdaaaiabgEna0kabigdaXiabicdaWmaaCaaabeqaaiabiAda2aaacqGGUaGlaaa@7147@

where *x *represented the normalized expression level of gene calculated by each platform. For MPSS platform, NEU has the same meaning as Transcript Per Million (TPM).

Contour-plots were used to represent the density of data. Expression data were *log*_2_-transformed. When investigating the correlation relationship between different samples, hierarchical clustering method was used with both Pearson's correlation and Spearman's rank correlation as the distance metrics and using a complete linkage algorithm [36,37]. The distance for clustering was calculated by 1 - *r*, where r represents correlation coefficient value. Correlation coefficient and total least-square values [38] were calculated for measuring the relationship among data from different platforms. A contour-plot matrix [25] was used to show correlation relationships. Because the relationship between two variables was often non-linear, we used a regression method called LOWESS (local weighted scatter-plot smoother) [39] to obtain smooth curves which showed the mean tendency of dependence pattern between two variables. When using LOWESS, we used a Gaussian kernel to calculate the weight contributing to each point from its neighboring points. We also used the bootstrap method [40] to evaluate the confidence intervals (5–95%) of the LOWESS method.

## Authors' contributions

VA and BM initiated the research, provided the MPSS data and finished the first version of analysis on the measurement abundance and ratio comparisons. JC improved the analysis and expanded it to the analysis of the probe-position effect in Affymetrix and tag-position effect in MPSS. JC also evaluated the results and drafted the manuscript. MR helped with the evaluation of the results and manuscript preparation, and provided mentoring. MW provided the Affymetrix experiments and data. RM, MW and DS provided input on experiment design, analysis ideas and Affymetrix data. SC provided the Agilent data. All authors read and approved the final manuscript.

## Supplementary Material

Additional File 1 **Supplementary Figures**. An additional pdf file includes supplementary figures. Click here for file
