# Supplementary material for: A comparison of microarray and MPSS technology platforms for expression analysis of Arabidopsis
Source: BMC Genomics. 2007 Nov 12;8:414. doi: 10.1186/1471-2164-8-414 (PMC2190774; doi:10.1186/1471-2164-8-414)
Supplement: Additional File 1 — Supplementary Figures. An additional pdf file includes supplementary figures. [file 1471-2164-8-414-S1.pdf]

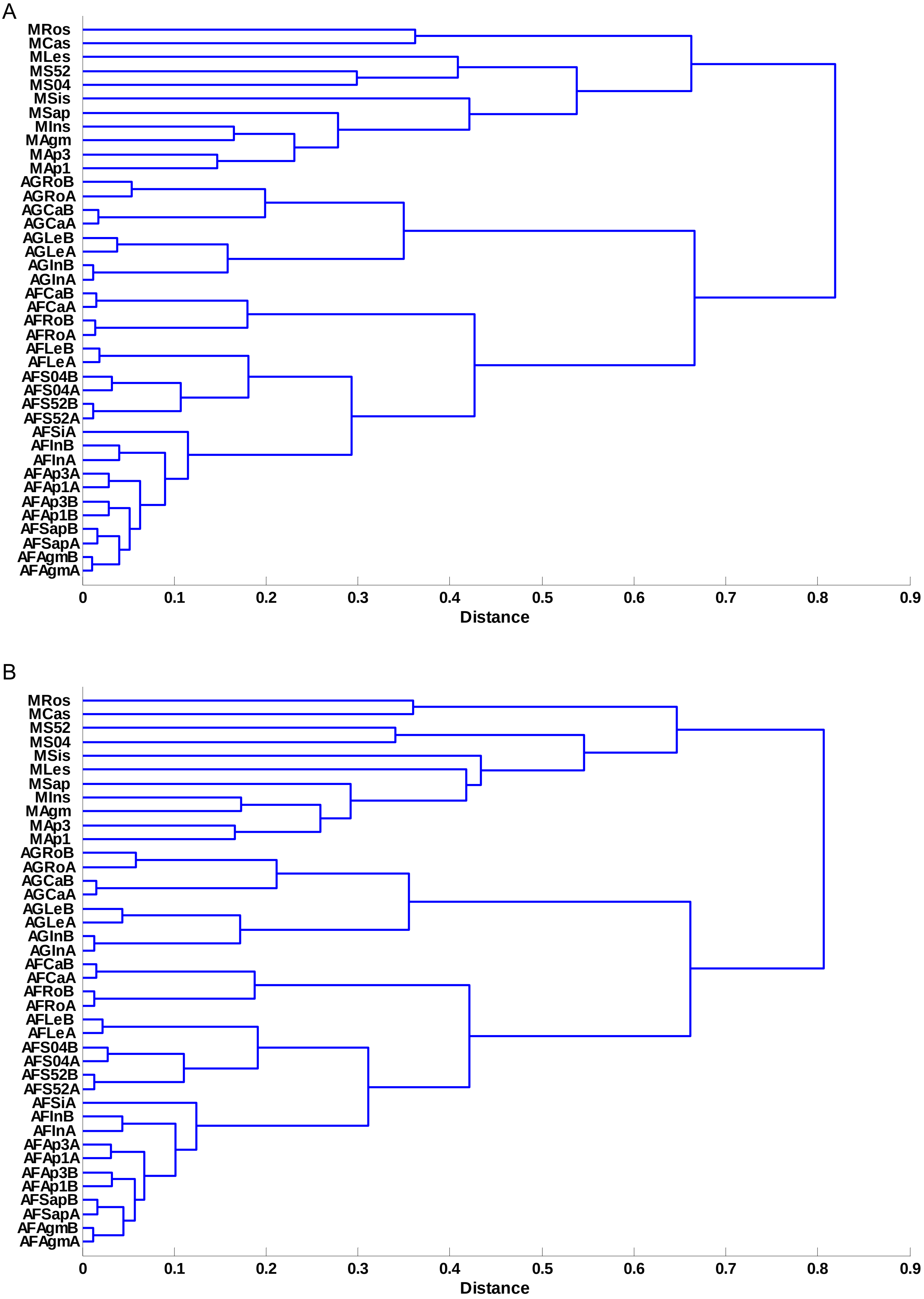

Figure 1 - Hierarchical Clustering of Correlations of Log(NEU) Data for 1,648 Genes in MPSS, Affymetrix and Agilent

Plot A shows the Pearson's correlation while Plot B shows the Spearman's rank correlation. Distances for clustering were calculated by 1-r, where r represents correlation coefficient value. Complete linkage algorithm was used for hierarchical clustering. All genes are selected with MPSS>=4 TPM, Affymetrix present detected in all samples.

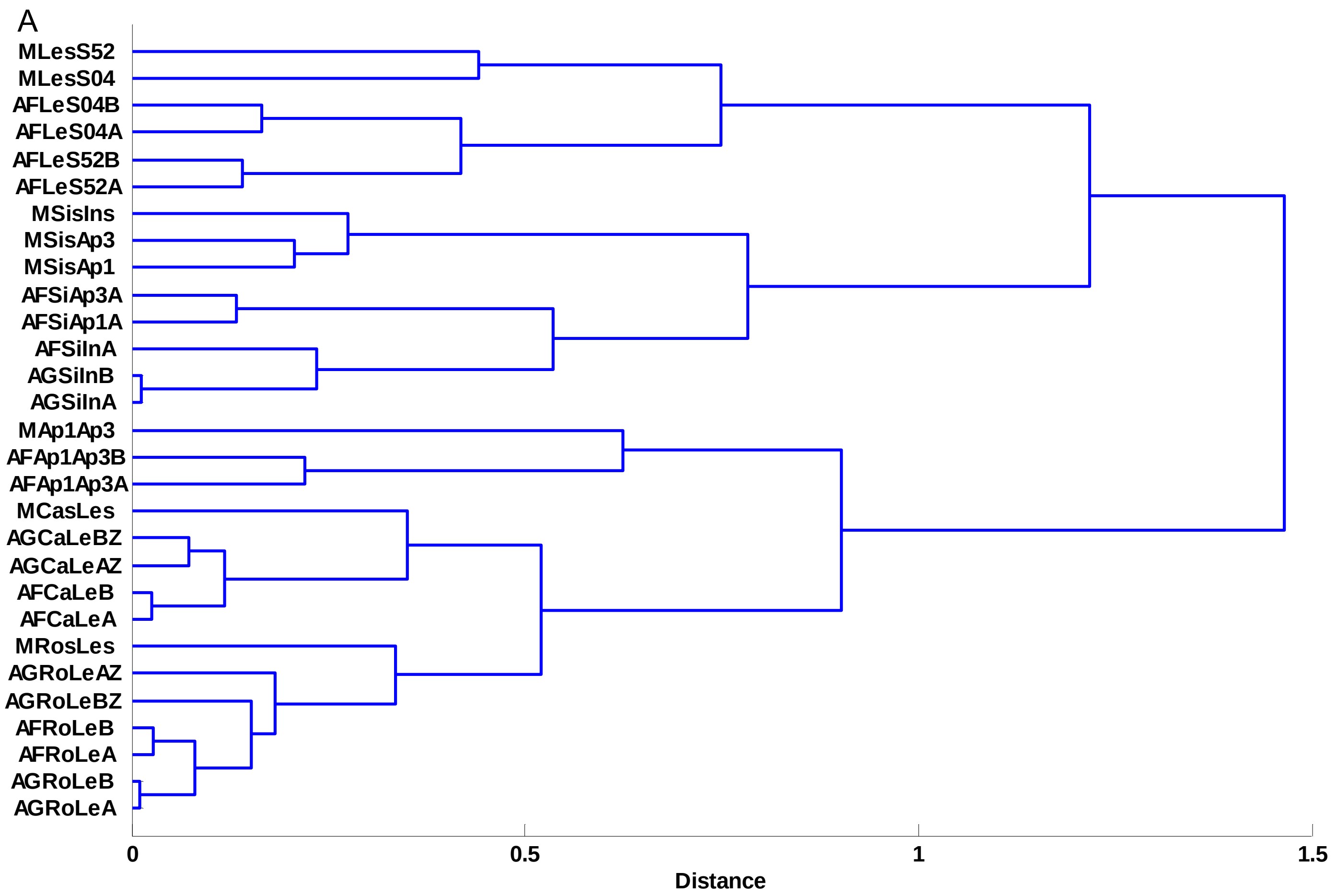

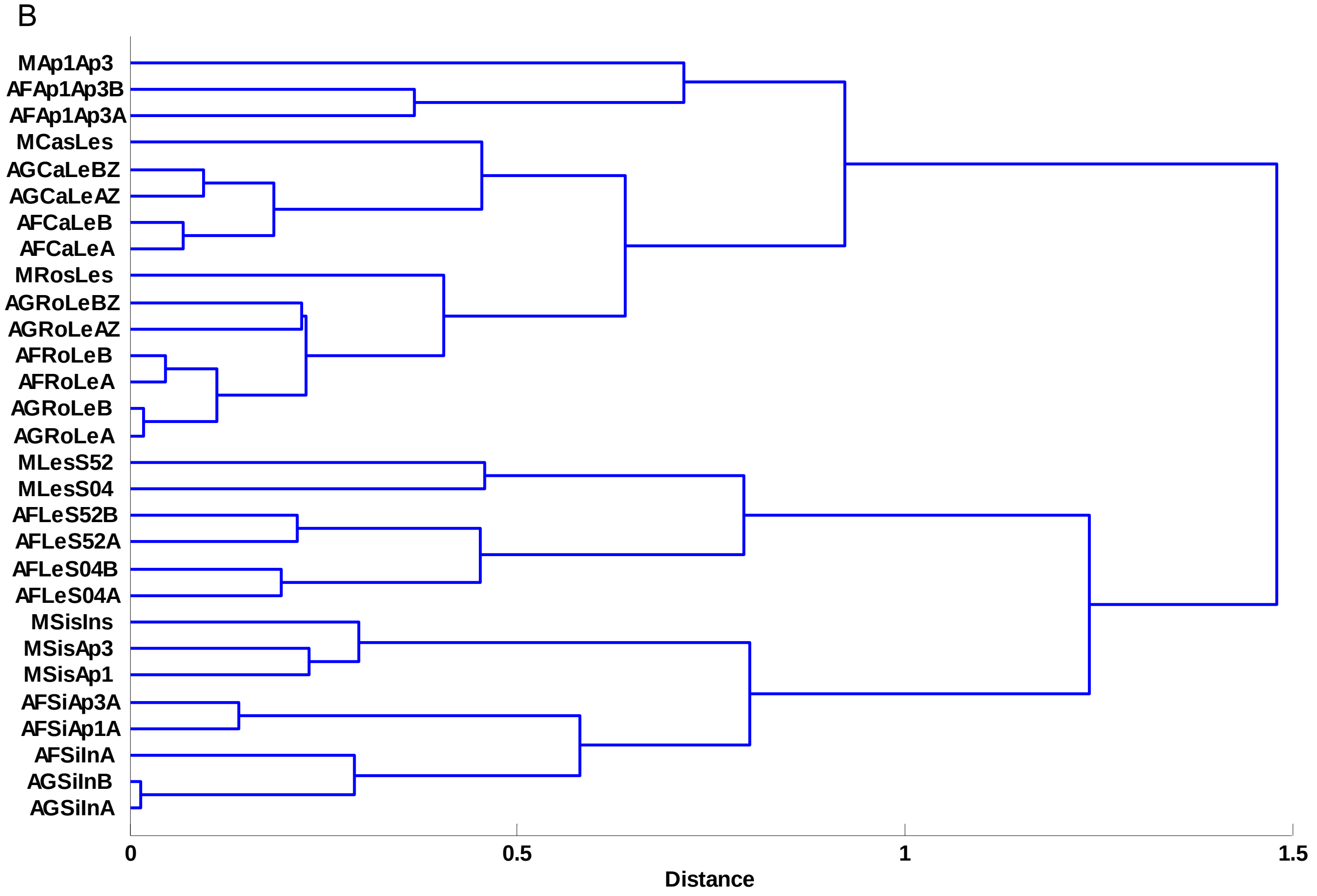

Figure 2 - Hierarchical Clustering of Correlations of Ratios Expression Data for 1,648 Genes in MPSS , Affymetrix and Agilent

Plot A shows the Pearson's correlation while Plot B shows the Spearman's rank correlation. Distance for clustering was calculated by 1-r, where r represents correlation coefficient value. Complete linkage algorithm was used for hierarchical clustering. Gene are the same with those in Figure 1.
